# Oxidative stress response of *Saccharomyces cerevisiae* exposed to different molecular weight of polycyclic aromatic hydrocarbons

**DOI:** 10.3389/fmicb.2026.1832345

**Published:** 2026-05-25

**Authors:** Pennapa Takam, Wasin Charerntantanakul, Panwad Sillapawattana

**Affiliations:** 1 Program in Biotechnology, Faculty of Science, Maejo University, Chiang Mai, Thailand; 2 Program in Environmental Technology, Faculty of Science, Maejo University, Chiang Mai, Thailand

**Keywords:** biomarker, eukaryote model organism, oxidative stress, PAHs, toxic effect

## Abstract

Polycyclic aromatic hydrocarbons (PAHs) are environmental contaminants that cause adverse health effects. This study investigated the toxic effect of different molecular weight PAHs on eukaryote model organism (*Saccharomyces cerevisiae*) exploiting oxidative stress biomarkers as endpoint indicators. Yeast cells were treated with environmentally relevant concentrations of phenanthrene (PHE), fluoranthene (FLA), and benzo[*ghi*]perylene (BghiP) for 24 h. Benzo[*a*]pyrene (BaP) was used as a positive control. Subsequently, expression of genes related to oxidative stress responses, cytotoxicity via targeted analysis of antioxidant mutants, generation of reactive oxygen species (ROS), superoxide dismutase (SOD) activity, and glutathione (GSH) content were assessed. The results demonstrated that viability of *S. cerevisiae* was related to the molecular weight of PAHs. Oxidative stress related genes *SOD1* and *SOD2* were found to be prominently expressed. According to the redox mechanism of action, BghiP and BaP triggered ROS formation and induced an increasing activity of SOD. Furthermore, PAH exposure significantly affected the glutathione levels. In summary, PAHs clearly caused oxidative stress. Among PAH samples, the more benzene ring contained more toxicity level exerted in yeast cells.

## 1 Introduction

Polycyclic aromatic hydrocarbons (PAHs) are one of the most significant causes of environmental pollution (Ismail et al., 2022). PAHs are produced both naturally and anthropogenically. Natural sources include forest fires, volcanic eruptions, tree exudates (pine needles), and oil seeps (Sakshi and Haritash, 2020). Anthropogenic sources of PAHs include the combustion of fossil fuels, coal tar, crude oil or petroleum spills, municipal solid waste incineration, vehicle emissions, petroleum refinery effluent, smoke from wood-burning stoves, and manufactured gas plants (coal gasification). Anthropogenic activities are primarily responsible for the contamination by PAHs in the environment. Ambient air is one of the major routes of long-term human PAH intake (Samburova et al., 2017). Particulate matter (PM) in the atmosphere is frequently an accumulation site for PAHs due to the small particle size and large specific surface area (Künzli et al., 2006; Yu et al., 2022). Moreover, PAHs can be found in the surface water and sediment due to drainage and deposition of suspended air particles (Ranjan et al., 2012). Up to 15 times of PAH concentration could be detected in biota in comparison to sediment due to bioaccumulation (Froehner et al., 2018). It is reported that PAHs from different sources and geographical regions have different potentially harmful characteristics (Byeong Kyu and Van Tuan, 2010).

As several PAHs generate inflammation, genotoxicity, mutagenicity, and carcinogenesis (Gad and Gad, 2014; Kim et al., 2013), the US Environmental Protection Agency (EPA) designated some of them as high-priority pollutants (He et al., 2022). Furthermore, the International Agency for Research on Cancer (IARC) classified PAHs into three categories: carcinogenic to humans, probably carcinogenic, and potential carcinogens (Ahmad, 2022). PAHs are categorized in three groups based on their molecular weight, including low-molecular-weight (LMW) PAHs containing 2–3 rings in their structure (MW of approximately 152–178 gmol^−1^), medium-molecular-weight (MMW) PAHs consisting of 4 rings (MW of about 202 gmol^−1^), and high-molecular-weight (HMW) PAHs bearing 5 or more rings (MW equal to or greater than 250 gmol^−1^) (Table 1) (De Giovanni et al., 2022; Safo-Adu et al., 2023).

**Table 1:** Characteristics of polycyclic aromatic hydrocarbons employed in this study.

PAHs	Structure	M_w_ (gmol^−1^)	Number of aromatic rings	Classified by M_w_
PHE	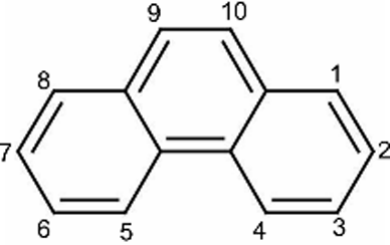	178.23	3	LMW
FLA	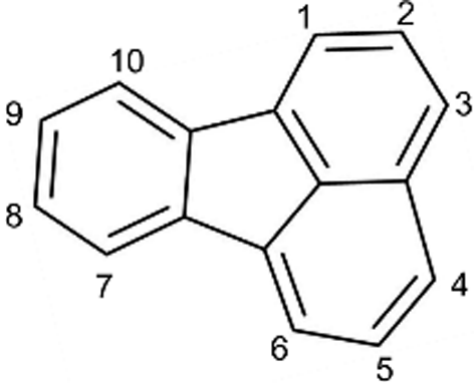	202.26	4	MMW
BghiP	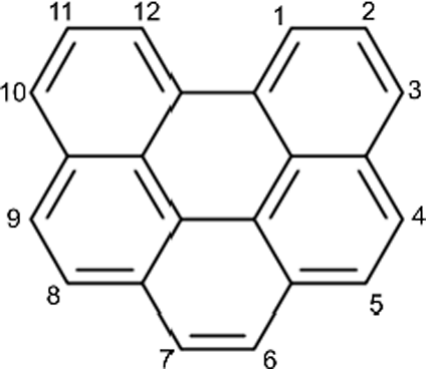	276.33	6	HMW
BaP	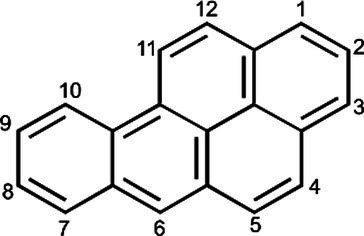	252.32	5	HMW

PAH-mediated conditions, such as cancer, are associated with their potential to indirectly induce genotoxicity via oxidative stress and generate adducts with DNA. Oxidative lesions and DNA damage are prominent hallmarks of PAH exposure (Adegbola and Adetutu, 2024). An imbalance between oxidants and antioxidants that impairs redox signaling causes molecular damage (Halliwell and Gutteridge, 2015). As an oxidative stress response of organism is a biomarker of exposure to some xenobiotics (Sillapawattana et al., 2024). Therefore, the oxidative stress pathway is useful for investigating exposure to ambient pollution (Peters et al., 2021; Wu et al., 2022) and assessing environmental risk. Oxidative stress biomarkers are classified into two categories: molecules that are impacted by interactions with reactive oxygen species (ROS) in the microenvironment and antioxidant system related molecules that change in response to an increased redox stress (Ho et al., 2013). In fact, oxidative stress is caused by the accumulation of ROS, which elevates the activity of antioxidant related enzymes such as superoxide dismutase (SODs), catalase (CTT) and enzymes of glutathione synthesis (GSH1 and GSH2) (Farrugia and Balzan, 2012).

The previous study investigated the toxic effects of three less investigated PAHs bound particulate matters with distinct molecular weights on co-culture model of human epithelial lung cells (A549) and macrophages (THP-1). The negative effects on A549 and THP-1 co-culture model implied an adverse effect on human health when coming into contact with these chemicals (Takam et al., 2024). In addition to the above mentioned study, yeast model has been employed in the present investigation as it is more practical for high-throughput toxicity testing than informative aforementioned approach (Walker et al., 2012; Wang et al., 2020). The responses of eukaryotic model organisms, *Saccharomyces cerevisiae*, to oxidative stress have been documented and applied as a biomarker to evaluate the toxic effect of xenobiotics in the environment (Sillapawattana et al., 2024). Due to the similarity between *S. cerevisiae* and mammalian cells, for instance, at the molecular and organelle levels, a number of yeast proteins have been found to be functionally interchangeable with human proteins (Costa and Moradas-Ferreira, 2001). Furthermore, yeast model is one of the most prominent experimental models for studying oxidative stress and its effects in the context of programmed cell death and ageing (Farrugia and Balzan, 2012). Therefore, the aim of this study is to evaluate the toxic effect of small, medium and high molecular weight groups of less investigated PAHs frequently detected in the environment on *S. cerevisiae* using oxidative stress responses as biomarker.

## 2 Materials and methods

### 2.1 Chemicals

Chemical used in this experiment, including phenanthrene (PHE) (MCE, USA, ref. HY-B1727), fluoranthene (FLA) (Sigma, China, ref. 206–44-0), benzo[*ghi*]perylene (BghiP) (Sigma-Aldrich, USA, ref. 191–24-2), and benzo[*a*]pyrene (BaP) (Sigma-Aldrich, USA, ref. 50–32-8), are categorized in three groups according to molecular weight. PHE, FLA, and BghiP represented: LMW, MMW, and HMW, respectively. BaP (HMW) was chosen as a positive reference for toxicology study (Table 1). PAH stock solutions were prepared in absolute dimethyl sulfoxide (DMSO) and placed in ultrasonic to ensure that chemicals were completely dissolved. Subsequently, desired test concentrations were prepared by diluting with culture medium. The highest concentration of DMSO used in this study was 2%. At this concentration, no effect on cell viability was observed (see Supplementary Figure 3).

### 2.2 Yeast strains and growth conditions

Wild-type yeast (BY4742: MATα; his3Δ1; leu2Δ0, lys2Δ0, ura3Δ0) and five mutant strains (Table 2) (Euroscarf, Germany) deficient in antioxidant related genes were maintained in YPD medium. The synthetic YPD medium was prepared by combining 10 gL^−1^ of yeast extract (Gibco, USA), 20 gL^−1^ of peptone (Gibco, USA), and 20 gL^−1^ of dextrose (VWR chemical, Belgium) incremented by 0.79 gL^−1^ of complete supplement mixture (CSM) (Himedia, India). The culture was incubated at 30 °C using shaking speed of 270 rpm (MIULAB, MU-E27-91030).

**Table 2:** The five mutant yeast isolates lacking the gene encoding the antioxidant related pathway.

Mutants	ORFs	Genotypes
gsh1Δ	YJL101c	MATα; his3Δ1; leu2Δ0, lys2Δ0, ura3Δ0; YJL101c::kanMX4
gsh2Δ	YOL049w	MATα; his3Δ1; leu2Δ0, lys2Δ0, ura3Δ0; YOL049w::kanMX4
sod1Δ	YJR104c	MATα; his3Δ1; leu2Δ0, lys2Δ0, ura3Δ0; YJR104c::kanMX4
sod2Δ	YHR008c	MATα; his3Δ1; leu2Δ0, lys2Δ0, ura3Δ0; YHR008c::kanMX4
ctt1Δ	YGR088w	MATα; his3Δ1; leu2Δ0, lys2Δ0, ura3Δ0; YGR088w::kanMX4

Prior to the experiment, yeast was grown in YPD broth at 30 °C for 24 h using a shaker speed of 270 rpm. For each experiment, yeast suspension was adjusted to an optical density of 0.2 by the same medium. The ratio of PAHs solution to yeast suspension was 7:3 (v/v).

### 2.3 Toxicity testing by chemo-genetic screening

The Chemo-genetic screening method was done as described by Sillapawattana et al. (2024). Briefly, yeast isolates were cultivated overnight at 30 °C using shaking speed at 270 rpm. After incubation, the yeast suspension was adjusted to an optical density at 600 nm (OD_600_) of 0.2 and exposed to various concentrations of each chemical in a 7:3 (V/V) ratio. For the control group, yeast strains were incubated in medium without chemical exposure. The experiments were conducted in triplicate using a 96-well plate and incubated for 24 h at 30 °C and 270 rpm. After exposure, yeast vitality was assessed by spotting the cultures onto YPD agar and following subsequent colony growth. The toxic effects of chemicals on yeast growth were observed by measuring the absorbance at 600 nm in the 96 well plate using a microplate reader (BMG LABTECH, USA).

### 2.4 Gene expression analysis by qPCR

For gene expression studies, yeast strain BY4742 was exposed to PHE, FLA and BghiP for 24 h at 30 °C in microtiter plates using shaking speed at 270 rpm. For each PAH, 3 concentrations were employed, i.e., 0.25, 0.5, and 0.75 mM. An expression of genes involving in antioxidant pathway, including *SOD1*, *SOD2*, *CTT1*, *GSH1*, and *GSH2* were studied. The details of primer used for qPCR are listed in Supplementary Table 1.

After 24 h-exposure, cells from the control and PAH-treated samples were collected and harvested by centrifugation. Subsequently, cells were broken with a homogenizer for 1 min on ice. Total RNA was isolated using the SV Total RNA Isolation System (Promega, USA) followed by the quantification of total RNA in a micro-volume spectrophotometer (Biometrics, Taiwan). For cDNA synthesis, equivalent amount (2 ngμL^−1^) of total RNA was reverse-transcribed to cDNA using a ReadyScript™ cDNA Synthesis Mix (Sigma, USA) according to the manufacturer’s instructions.

qPCR was conducted using 2X SYBR green (biotechrabbit™, Germany) master mixes. The thermal cycler (QIAquant 96 5plex, Qiagen, Germany) condition was set as follow: an initial denaturation at 95 °C for 3 min, 45 cycles of denaturation at 95 °C for 15 s and annealing at 62 °C for 30 s. Melting curve analysis was performed for each primer pair. The 2^-ΔΔCT^ method (Livak and Schmittgen, 2001) was used to quantify the relative changes in expression levels between control and treatment samples. The relative expression fold change was normalized by comparison with the housekeeping gene, *actin 1* (*ACT1*).

### 2.5 Oxidative stress response of yeast cells exposed to PAHs

Yeast culture BY4742 was adjusted to an OD_600_ of 0.2 and treated with three different concentrations of each PAH on 24 well-plates, then incubated for 24 h at 30 °C using a shaking speed of 270 rpm. Each test condition was done in triplicate. Subsequently, cells were harvested by centrifugation (Ugaiya Biosciences, Japan) at 4,000 rpm for 10 min and kept for further experiments.

#### 2.5.1 ROS assay

Cells were washed with 143 mM PBS buffer containing 63 mM EDTA and resuspended by the same buffer. 95 μL of cell suspension from each test condition was added to black flat-bottom 96-well plates. Then, 5 μL of 10 mM H_2_DCFDA (Invitrogen, USA) was added to each well and incubated at room temperature for 30 min. Later, fluorescent signal was detected (excitation at 490 nm and emission at 520 nm) using a fluorescence microplate reader (Synergy H1, BioTek, USA). H_2_DCFDA was used as an indicator for reactive oxygen species (ROS) in living cells. Upon cleavage of the acetate groups by intracellular esterases and oxidation, the nonfluorescent H_2_DCFDA was converted to the highly fluorescent 2′,7′-dichlorofluorescein (DCF). The formation of ROS in living cells exposed to PAHs was expressed as relative fluorescence unit (RFU).

#### 2.5.2 Determination of SOD activity

Cells from control and PAHs treated groups were simultaneously extracted according to the method described by Sillapawattana et al. (2024). Briefly, harvested cells were rinsed with 143 mM phosphate buffer pH 7.5 containing 63 mM EDTA and centrifuged (Ugaiya Biosciences, Japan) at 4 ^o^ C for 5 min at 4,000 rpm to decant buffer. This step was repeated two times to complete the total rinsing of 3 times. Subsequently, the cell pellets were resuspended in 5 volumes (w/v) of phosphate buffer. Later, the cells were broken by vortexing with glass beads for 1 min and placed on ice for 1 min. This manner was repeated 2 more times. In order to remove the glass beads, the samples were centrifuged at 4^o^ C, 4,000 rpm for 10 min and the supernatant was collected and immediately analyzed to prevent the loss of enzyme activity or otherwise stored at −80 °C until use. SOD activity was measured *in vitro* using a SOD measurement kit (Sigma-Aldrich, Germany) by indirect method using the water-soluble tetrazolium salt that produces a water-soluble formazan dye upon reduction with a superoxide anion. The rate of the reduction with O_2_ is linearly related to the xanthine oxidase (XO) activity, and is inhibited by SOD. Since the absorbance at 440 nm is proportional to the amount of superoxide anion. The SOD activity was expressed as an inhibitory activity that could be evaluated by detecting the decrease in color development at 450 nm using the manufacturer’s procedure.

#### 2.5.3 GSH assay

Harvested cells were washed with 143 mM phosphate buffer pH 7.5 containing 63 mM EDTA and centrifuged for 5 min at 4,000 rpm. This step was repeated two times. Cells were then weighed and resuspended in 3 volumes (w/v) of 5% salicylic acid solution. Later, the cells were broken by vortexing with glass beads for 1 min and placed on ice for 1 min. This procedure was repeated three times. Glass beads were removed from the samples by centrifugation at 4000 rpm for 10 min. The supernatant was collected and stored at −80 °C until use. The measurement of glutathione content was performed on 96-well plate by enzymatic recycling reaction according to the manufacturer’s protocol with slight modification (CS0260, Sigma-Aldrich, USA). In brief, GSH was oxidized to GSH disulfide (GSSG) by 5,5′-dithiobis-(2-nitrobenzoic acid) (DTNB) and GSSG was then catalyzed by GSH reductase (Sigma, USA) in the presence of NADPH (Sigma, USA). The formation rate of the yellowish substance, 2-nitro-5-thiobenzoic acid (TNB), was proportional to the amount of GSH and was monitored at 412 nm over the suitable period (linear progression curve). The total GSH amount was determined from the standard curve of reduced glutathione.

### 2.6 Statistical analysis

The statistical data was analyzed using SPSS V.29. The differences were assessed using one-way analysis of variance (ANOVA). Tukey’s HSD was used to analyze the difference between groups. All experiments were done using 3 biological replicates and for each condition, the test was done in triplicate.

## 3 Results and discussion

### 3.1 Targeted analysis of antioxidant mutants

**Figure 1:**
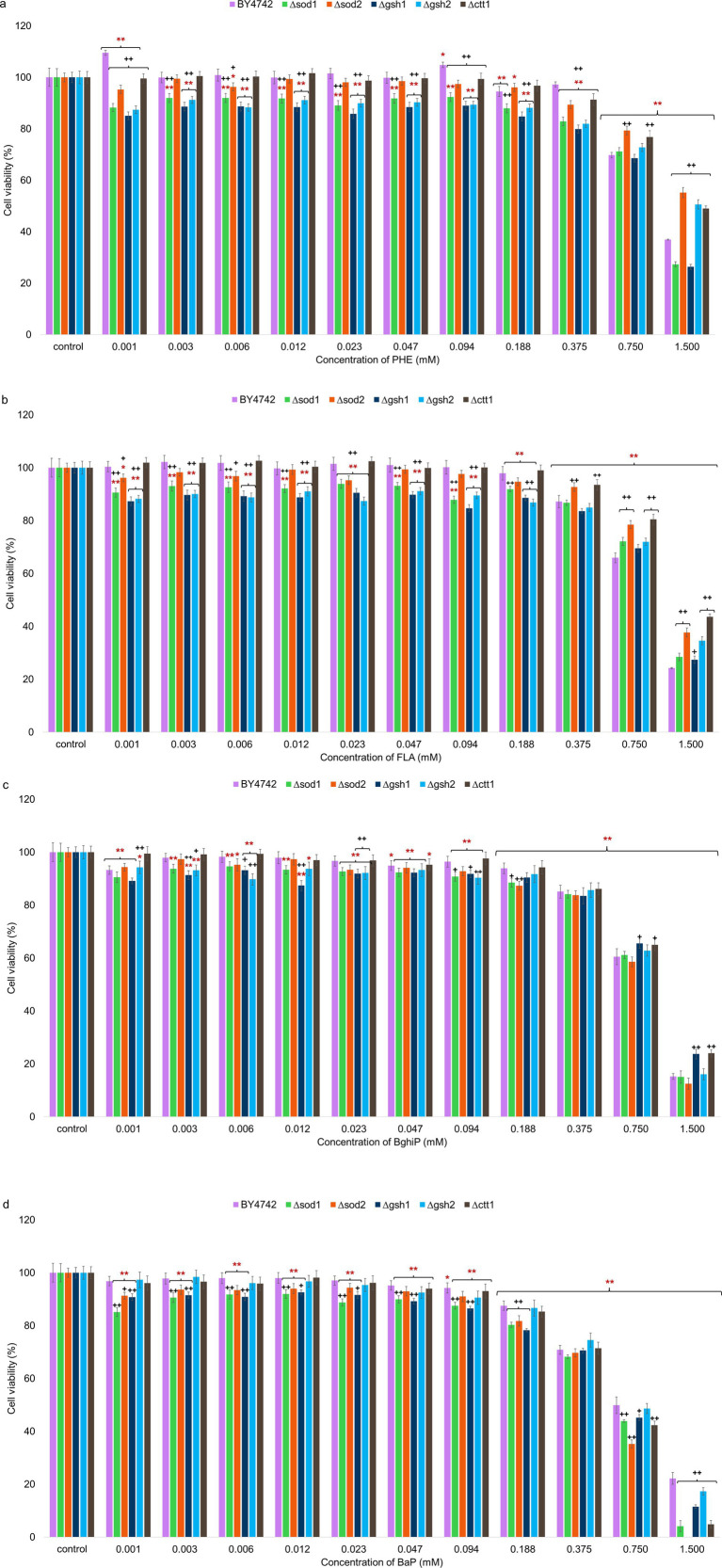
Toxic effect of different concentrations of PAHs on BY4742 and five mutants. **(a)** LMW PAHs (PHE), **(b)** MMW PAHs (FLA), **(c)** HMW PAHs (BghiP), and **(d)** positive control (BaP). The statistical comparison between mutants in response to each PAH is represented as + and ++ when *p* < 0.05 and 0.01, respectively. The statistical comparison between different concentration of each PAH and control is illustrated as * and **, when *p* < 0.05 and 0.01, respectively.

Analysis of mutants impaired in antioxidant defense pathway was performed to assess the toxicity of PAHs on wild-type BY4742 and five mutants lacking gene encoding antioxidative stress related enzyme, including mitochondrial and cytoplasmic superoxide dismutase, catalase, *γ*-glutamylcysteine synthetase, and glutathione synthetase (for detail see Table 2). The effect of PAHs on the growth rate of the test organisms is shown in Figure 1.

As shown in Figure 1, the survival rate of yeast cells was dependent upon the exposure concentration. Namely, the survival rate of test organisms decreased when the PAHs concentration increased in comparison to the untreated group. Among the yeast strains tested, sod1Δ and sod2Δ mutants were the most susceptible to BghiP and BaP, indicating elevated level of ROS formation caused by both chemicals in the cells. This is because SODs are the first line of defense against oxygen-free radicals produced by organisms living in the presence of oxygen to eliminate excess ROS and restore redox stability. Both mitochondrial SOD (MnSOD) and cytosolic SOD (CuSOD) scavenge O_2_^•-^ and convert it to H_2_O_2_, which is subsequently degraded to water and oxygen by cytosolic catalase (CTT1) (Farrugia and Balzan, 2012). Therefore, mutants lacking *SOD* may exposed to ROS accumulated in the cells during exposure to BghiP and BaP, which diminished survival.

As seen from Figure 1, five-ring containing PAH (BaP) and six-ring containing PAH (BghiP) are more toxic to wildtype and mutants than three-ring containing PAH (PHE) and four-ring containing PAH (FLA). This reflects the significance of molecular weight on toxicity, by which HMW-PAHs are substantially more toxic than LMW-PAHs. In agreement with the results from other studies conducted in a cell culture model, Ke et al. (2018) investigated the effects of 16 priority PAHs on human pulmonary alveolar epithelial cells (HPAEpiC). The results showed that PAHs with 5-ring structures (BkF, BaP, BbF, and DBA) and 6-ring structures (IND, BghiP) exhibited significant cytotoxicity toward HPAEpiC. Previous studies have also demonstrated that BghiP (a 6-ring PAH) and BaP (a 5-ring PAH) can induce oxidative stress and apoptosis by activating the intracellular aryl hydrocarbon receptor (AhR) pathway (Cherng et al., 2001; Yu et al., 2008; Zaragoza-Ojeda et al., 2016).

In order to distinguish lethal and inhibitory concentrations of PAHs, the vitality of *S. cerevisiae* was assessed by colony formation on YPD agar (Supplementary Figure 2). Based on the results in this part, exposure concentrations of 0.25, 0.5, and 0.75 mM, were selected to perform further experiments. As PAHs concentrations found in the environments are varied, in order to keep the test concentrations to be closely to the environmentally relevant one as much as possible, the test ranges leading to more than 80% viability of cell culture from the previous work (Takam et al., 2024) together with the ones found in PAHs polluted surface water and sediments were employed (Froehner et al., 2018).

### 3.2 Gene expression

**Figure 2:**
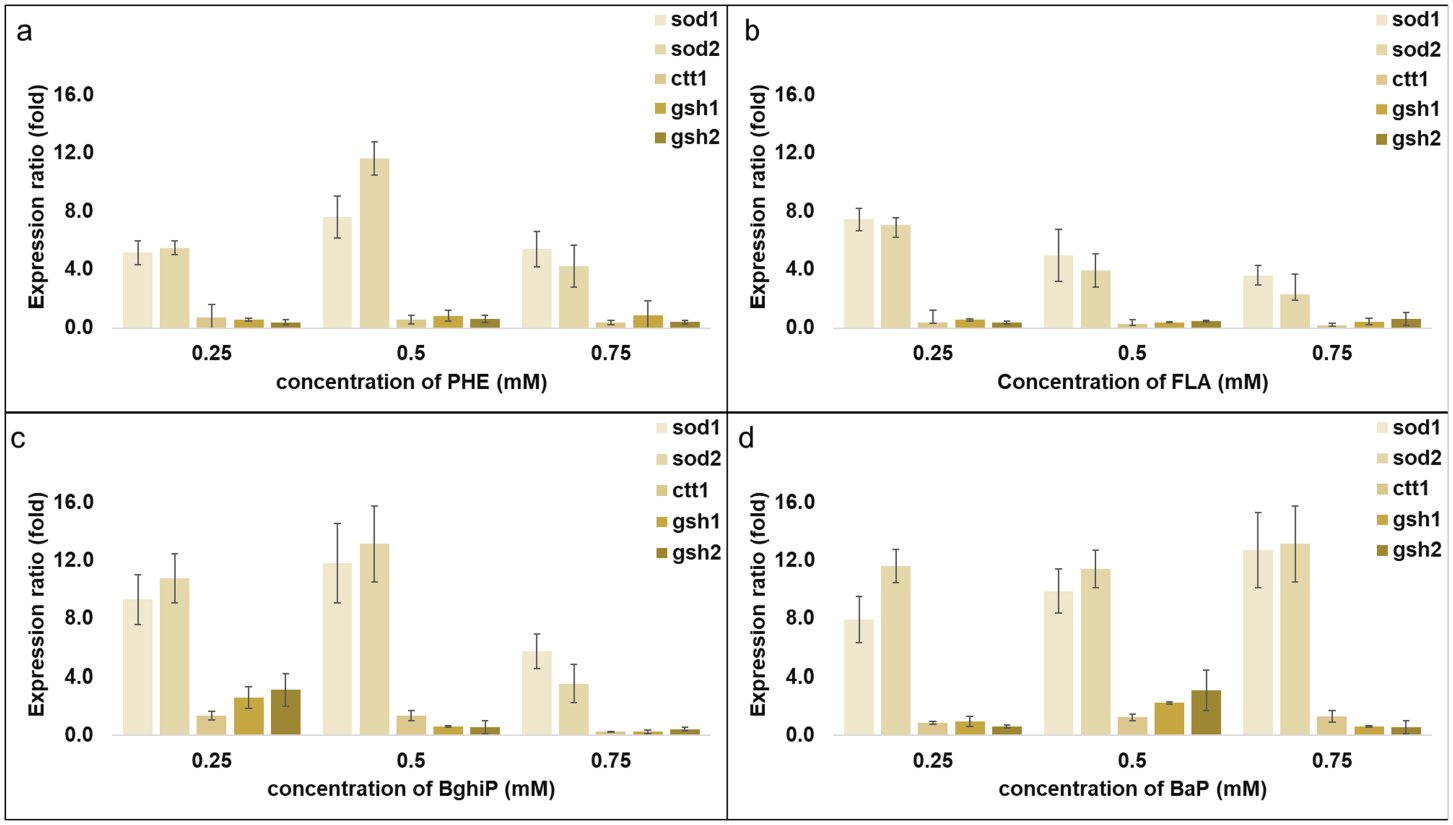
Mean steady-stage mRNA level of target genes from yeast BY4742 exposed to PAHs for 24 h normalized by reference gene *ACT1* (beta-actin) using the 2^-ΔΔCt^ method. **(a)** LMW PAHs (PHE); **(b)** MMW PAHs (FLA); **(c)** HMW PAHs (BghiP); and **(d)** positive control (BaP).

Gene expression analyses were used to investigate the signaling and metabolic pathways that regulate oxidative stress response of yeast cells to PAHs. The qPCR amplification efficiency for 6 genes ranged from 91.60 to 106.22%, the melting temperature was between 82.9 and 89.0 °C, and the correlation coefficients were between 0.8919 and 0.995 (Supplementary Table 1). The mRNA steady-state levels of BY4742 exposed to different molecular weight groups of PAHs for 24 h was illustrated in Figure 2.

From the results, exposure to PHE, FLA and BghiP showed higher expression level of *SOD1* and *SOD2* than those of *CTT1*, *GSH1*, and *GSH2*. For each PAH treated group, the expression level of *SOD1* and *SOD2* were decline at the highest concentration (0.75 mM). In case of positive control (BaP treated cells), *SOD1* and *SOD2* were highly expressed in every test concentration.

At the concentration of 0.25 mM, the mean expression levels of *SOD1* and *SOD2* from test organisms exposed to BghiP were the highest, followed by FLA and PHE, respectively, as illustrated in Figure 2. The mean expression levels of *SOD1* in yeast upon exposure to different concentrations of BghiP, FLA, PHE, and BaP were approximately 9.29 ± 1.72, 7.43 ± 0.77, 5.14 ± 0.83, and 7.91 ± 1.58 fold, respectively. Similarly, the mean expression of *SOD2* in yeast exposed to BghiP, FLA, PHE, and BaP was upregulated by 10.73 ± 1.69, 7.05 ± 0.84, 5.46 ± 0.48, and 11.61 ± 1.14 folds, respectively. Furthermore, an alteration of the mean steady state level of target genes was detected to be higher in test organisms exposed to HMW-PAHs, i.e., *CTT1* (1.34 ± 0.29 fold), *GSH1* (2.60 ± 0.76 fold), and *GSH2* (3.12 ± 1.12 fold) than those exposed to MMW and LMW PAHs. The results at the molecular level revealed that HMW-PAHs may possibly up-regulate genes related to an oxidative response. Subsequently, further research was conducted at the cellular level to determine the response of oxidative stress biomarker to PAHs by evaluating the redox mode of action of PAHs in yeast.

### 3.3 Oxidative stress response of yeast cells exposed to PAHs

#### 3.3.1 ROS assay

**Figure 3:**
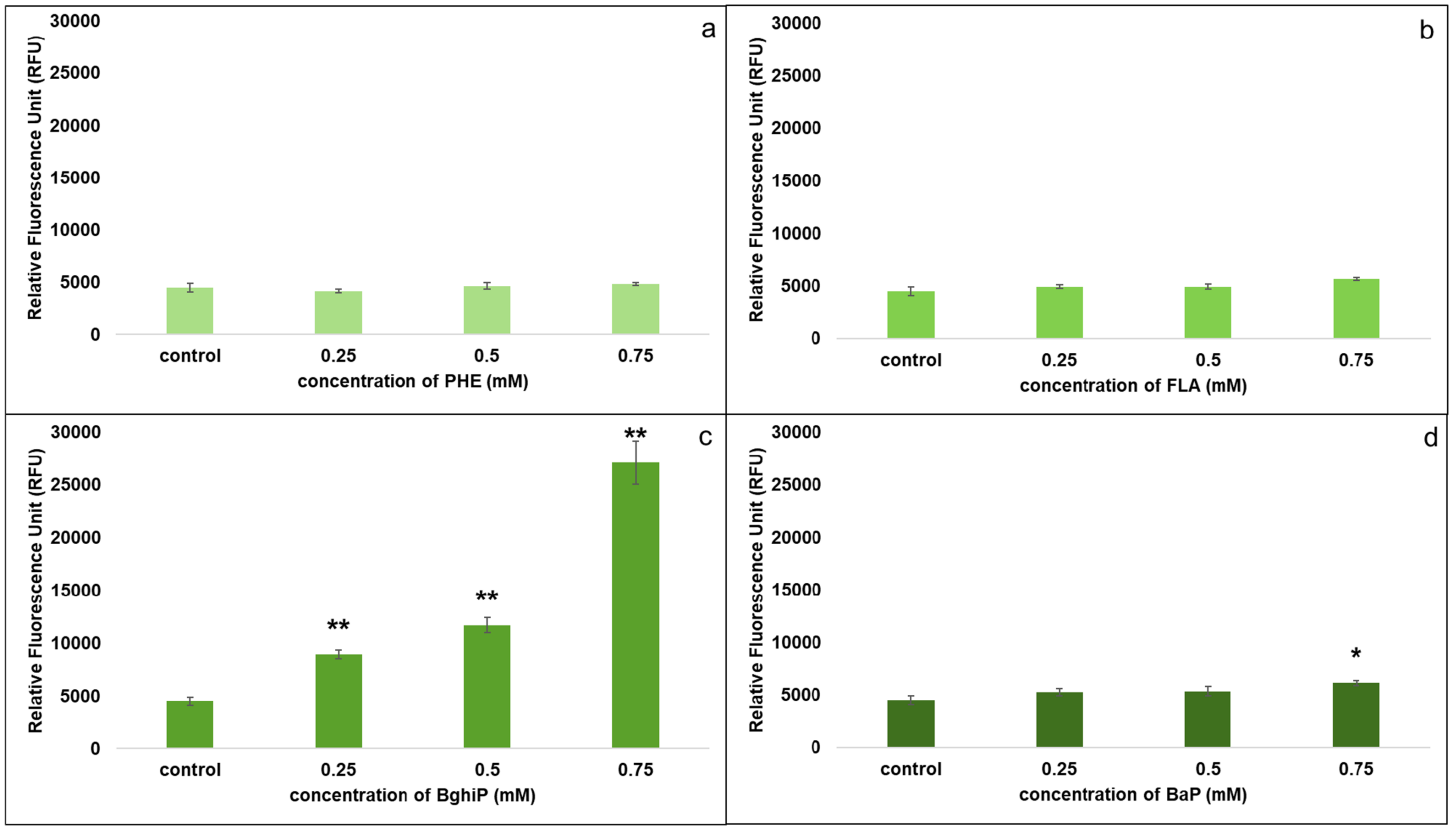
Relative fluorescence units indicating ROS formation in yeast BY4742 exposed to PAHs for 24 h. **(a)** LMW PAHs (PHE); **(b)** MMW PAHs (FLA); **(c)** HMW PAHs (BghiP); and **(d)** positive control (BaP). Single and double asterisks indicate significant levels for *p* < 0*.05* and *p* < 0.01, respectively.

ROS formation in BY4742 exposed to PAHs was measured using the specific dye H_2_DCFDA as illustrated in Figure 3. The amount of intracellular ROS formation in yeast cells treated with PHE and FLA was similar to the untreated group. However, BghiP exposure enhanced oxygen radical production. From the result, it is clearly revealed that HMW PAH caused a stronger degree of ROS formation than the MMW and LMW PAHs in yeast cells. However, low level of ROS generation of yeast cells treated with BaP, was detected, though this PAH contains 5 aromatic rings.

#### 3.3.2 Determination of SOD activity

**Figure 4:**
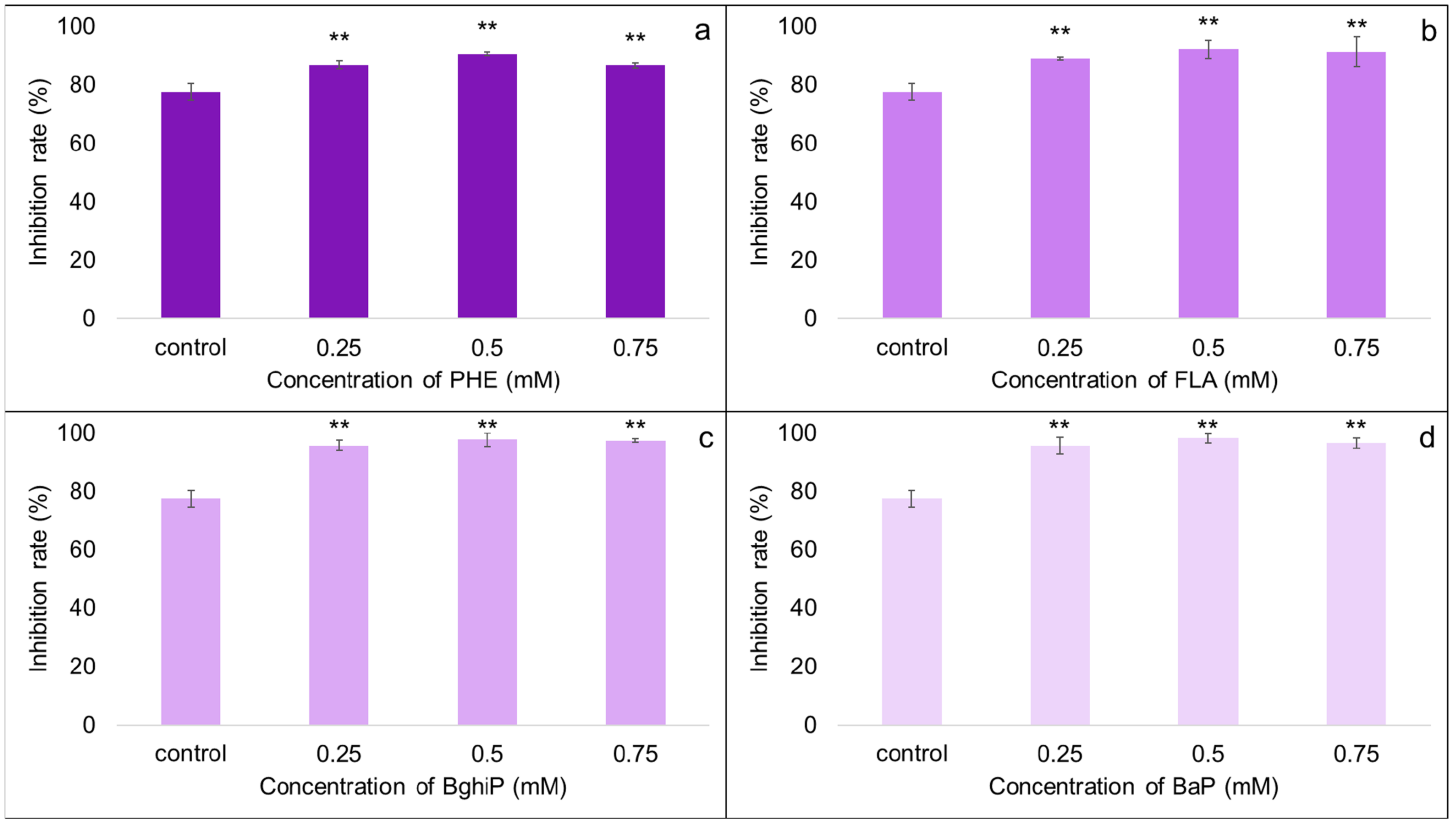
SOD activity of BY4742 exposed to PAHs for 24 h. **(a)** LMW PAHs (PHE), **(b)** MMW PAHs (FLA), **(c)** HMW PAHs (BghiP), and **(d)** positive control (BaP). Double asterisk indicates significant level for *p* < 0.01.

The activity of SOD in BY4742 exposed to PAHs was reported as % inhibition as displayed in Figure 4. From the results, intracellular SOD activity of organisms treated with 0.25–0.75 mM of each PAHs was significantly different from the untreated group. As SOD is the first line of enzymatic defense against oxygen free radicals, this points to the fact that PAHs cause ROS in yeast cells.

The expression levels of *SOD1* and *SOD2* were consistent with changes in SOD activity after PAH exposure. According to the results obtained, PAH up-regulated *SOD1* and *SOD2* expression levels and elevated SOD activity in yeast cells. These findings suggested that during BaP exposure, the respond at the transcriptional level may transfer to the translational or post-translational level.

#### 3.3.3 GSH assay

**Figure 5:**
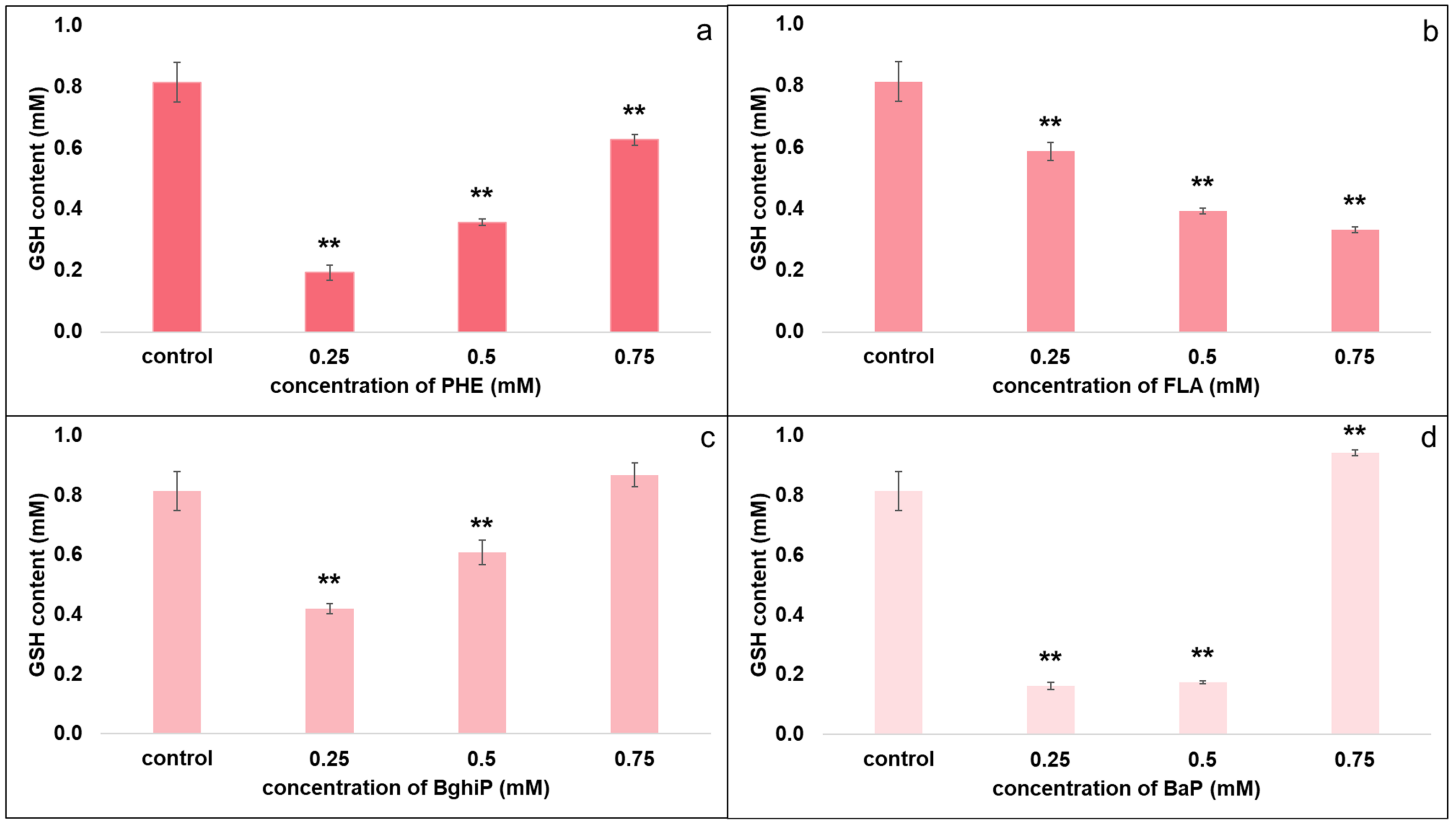
Glutathione content of BY4742 exposed to PAHs for 24 h. **(a)** LMW PAHs (PHE); **(b)** MMW PAHs (FLA); **(c)** HMW PAHs (BghiP); and **(d)** positive control (BaP). Double asterisks indicate significant level for *p* < 0.01.

The amount of GSH pool in BY4742 exposed to PAHs for 24 h was quantified and reported in Figure 5. The GSH content of the 0.25- and 0.5-mM PAH-treated groups were lower than the control group. However, at the highest concentration (0.75 mM) of BghiP and BaP-treated group, the GSH amount was slightly higher than those of the control group. In case of FLA treated group, total GSH amount was gradually decreased and reached the lowest level when exposed to the highest concentration of FLA. This is possibly due to the utilization of GSH to reduce the toxicity of FLA within the cells. In 0.25 mM of PHE- and BghiP-treated groups, the least amount of cellular GSH was detected, which was similar to the positive control group (BaP).

GSH is a low molecular weight thiol found in all aerobic cells and serves as a major non-protein antioxidant in the cells. The change of GSH level after PAH exposure may derive from many factors that come into play in order to protect cells from destructive effect of free radicals and detoxify environmental chemicals. The glutathione system comprising of GSH and two enzymes, i.e., glutathione peroxidase (GPx) and glutathione reductase (GR) forms the GSH redox system to keep the intracellular GSH homeostasis (Li et al., 2022). For this purpose, GSH is employed as substrate for peroxide detoxification, including hydrogen peroxide and lipid peroxides by GPs and subsequently transformed to oxidized GSH (glutathione disulfide: GSSG), which is in turn reduced to GSH by GR in the presence of NADPH (Griffith, 1999). Apart from enzymatic reaction, the protective function of GSH could spontaneously occur, since GSH is a strong nucleophile. Therefore, it can bind to electrophilic reactive compounds.

For screening step, the level of antioxidant form of glutathione (GSH) could tentatively indicate the state of oxidative stress of treated cells in comparison to untreated cells (control). In healthy cells high ratio of GSH to GSSG could be observed. However, in the present study, only the alteration of GSH was focused. The reduction of GSH levels may either point to the non-enzymatic usage of GSH to neutralize free radicals or GSH employment as substrate for the detoxifying process catalyzed by glutathione S-transferase. Both of which lead to the change of GSH level.

Overall experimental results demonstrated that PAHs could affect both molecular and cellular organization level of yeast cells. Namely, PAHs triggered ROS generation, altered the mRNA steady state level of oxidative stress related genes, especially *SOD1* and *SOD2,* resulting in an elevated level of SOD activity. Furthermore, PAHs could significantly affect the GSH content and cell viability. The toxic effects of PAHs on the test organisms were shown to be related to their molecular weight. As these contaminants cannot be easily degraded, their toxicity and persistence rise with an increase in molecular weight (Sakshi and Haritash, 2020). Generally, HMW PAHs are absorbed by sediments, whereas LMW PAHs can be present in the water in insignificant concentration (Froehner et al., 2018). In the atmosphere, LMW PAHs are more volatile and tend to remain in the gas phase. Although the LMW-PAHs molecules are considered less toxic, they are able to react with other pollutants (such as O_3_, NO, and SO_2_) to generate nitro- and dinitro-PAHs, and sulfuric acids, respectively. The toxicity of such molecules may be more significant than the parent compounds because of biotransformation (Kim et al., 2013; Park et al., 2001). Moreover, LMW-PAH such as PHE contain chemical structures, for instance bay and K regions, which are resemble carcinogenic HMW-PAHs found in BaP and benz[a]anthracene (Mallick et al., 2011). Therefore, the transformation of some PAHs may release more toxic products than parent PAHs and can lead to critical cellular effects (Ghosal et al., 2016). HMW-PAHs are more likely to cause cancer, whereas LMW-PAHs are more acutely toxic (Kim et al., 2013). This is because of the fact that the majority of the heavier PAHs (MMW and HMW-PAHs) are in the particulate phase in the atmosphere due to their low vapor pressure (Kameda et al., 2005). As reported by Kim et al. (2013), the percentages of PHE, FLA, BghiP, and BaP found in the particulate phase were 9, 16, 83, and 89, respectively. Ryu et al. (2023) documented an easily access of heavy particulate matter (PM) synthesized from carbon black and BaP to mitochondria, the primary site of reactive oxygen species (ROS) generation, thereby inducing cellular damage and apoptosis. PAHs are water insoluble and tend to accumulate in the fatty tissues of organisms, particularly in aquatic ecosystems, and may enter the food chain. This poses an increasing exposure risk to top predators, including humans (Padilla-Garfias et al., 2024). The log*K*_ow_ of PAHs range approx. From 3.37–7.66. LMW PAHs possess lower log*K*_ow_ than MMW and HMW PAHs. The log*K*_ow_ of PHE, FLA, BaP and BghiP are 4.57, 5.52, 6.04 and 6.50, respectively (LaGrega et al., 2010). Chemicals with high log*K*_ow_ (i.e., >4.57) are high bioavailability and thus are highly accumulated in living organisms. This is in line with our finding that the toxicity of PAHs is possibly related to the number of rings contained. Apart from chemical structure of PAHs, biotransformation of PAHs also comes in to play and should be considered.

The enzymatic detoxification of xenobiotics is classified into three distinct phases that act in an integrated manner. Phase I and II involve the conversion of a lipophilic, non-polar xenobiotic to more water-soluble forms, which are less toxic and are then excreted from the cells by phase III enzymes. Metabolic activation of BaP has long been studied. In yeasts, phase I biotransformation is significant, since the stable aromatic rings cannot be broken down before initial activation (Aranda, 2016). This metabolic activation is catalyzed by enzymes such as cytochrome P450 monooxygenase (CYPs) (Ostrem Loss and Yu, 2018; Padilla-Garfias et al., 2022), which oxidize the aromatic rings of PAHs, converting them into epoxides. Epoxides are reactive intermediates more easily metabolized in the subsequent phases of the degradation process (Padilla-Garfias et al., 2024). PAHs are frequently metabolized by cytochrome P450 monooxygenases (CYPs) and other metabolic enzymes into various substances such as phenols, catechols, and quinones, leading to the formation of diol epoxides (Liu et al., 2002). For example, biotransformation products of BaP such as 7,8-diol-9,10-epoxide (BPDE) is a strong carcinogenic chemical known to induce adverse effects on DNA, protein, and other macromolecules (Lee and Gu, 2003). Epoxide hydrolase catalyzes the addition of a water molecule to an arene oxide, resulting in a trans-dihydrodiol. Trans-9,10- and 4,5-dihydrodiols, as well as trans-7,8-dihydrodiol, are carcinogenic compounds generated by fungus in response to BaP exposure. Most PAHs contain complicated fused ring structures that allow metabolic production of more than one trans-dihydrodiol isomer (Cerniglia, 1984; Sutherland, 1992). Lee and Gu (2003) investigated the expression patterns of DNA damage checkpoint and cytochrome P450 genes, both of which are associated with carcinogenesis in higher eukaryotic systems, including budding yeast in response to BaP. For phase II, the compound is catalyzed by glutathione-S-transferase (GST), forming glutathione-conjugated compounds, which are less toxic and more soluble. For phase III: conjugated products are excreted or internalization into the vacuole, and assimilation by other organisms.

ROS concentration may be enhanced by chemical exposure (Halliwell and Gutteridge, 2015). The presences of xenobiotics and elevated ROS levels are factors in various pathologies (Lushchak, 2012). Principally, GSH may prevent the development of pathology either by direct interaction with xenobiotics or by enzymatic catalyzed manner. Some xenobiotics can be directly autoxidized leading to ROS production. However, most xenobiotics are not directly autoxidized and contribute to pathology after transformation via different mechanisms. Many substances are oxidized by wide-range of endogenous oxygenase with the production of ROS at this stage. Bio-transformed xenobiotics may also undergo autoxidation with concomitant ROS generation. In order to prevent this scenario, cells utilize phase II detoxification enzymes. At this stage, GSH either conjugates with xenobiotics or acts as a substrate for enzymatic catalyzed conjugation reactions (Stenersen, 2004). From the results presented, changes in GSH content are possibly due to oxidative stress defense by neutralization of ROS and GST activity during biotransformation process. Both of which lead to the change of GSH level.

The response mechanisms to oxidative stress in *S. cerevisiae* are regulated at the transcriptional level mainly by the transcription factors (TFs) Yap1p, Skn7p, Msn2p, and Msn4p. These transcription factors response to different oxidative stressors by suppressing or upregulating the transcription of specific genes, many of which are related to antioxidant defenses (Farrugia and Balzan, 2012). Msn2p and Msn4p (Msn2/4p) were found to modulate genes encoding antioxidant enzymes (*CTT1*, *SOD1*, *SOD2*, *PRX1*, and *TSA2*) to decrease stress caused by various factors by eliminating ROS. Yap1p in *S. cerevisiae* is a significant player in the cellular response to oxidative stress and xenobiotic insults (Lushchak, 2012) by controlling the expression of genes associated with the synthesis of stress proteins and antioxidants such as GSH, CuZnSOD and catalase (Halliwell and Gutteridge, 2015). During oxidation, cysteine residues in Yap1p are oxidized to disulfide bridge. This causes conformation changes, allowing it to migrate to the nucleus and leads to an increase in Yap1-regulated gene expression. Since the molecular and cellular responses to PAHs were regulated by TFs, oxidative stress biomarkers associated with Yap1p and Msn2/4p, such as SOD activity and GSH levels, can possibly be utilized to determine toxicity. Our results suggested that the alteration of the steady state level of *SOD* mRNA, SOD activity as well as GSH content may be involved in the response of *S. cerevisiae* to PAH exposure. All these molecular and cellular changes reflect the sensitivity of oxidative stress biomarker as early-stage assessment for chemical exposure (Sillapawattana et al., 2024). In agreement with our findings, the study of Yap1p knockout by Kuge and Jones (1994) reported the decrease in activity of enzymes involved in oxidative detoxification such as glucose-6-phosphate dehydrogenase, superoxide dismutase, and glutathione reductase when exposed to hydrogen peroxide and chemicals that generate superoxide anion radicals (Li et al., 2022).

PAHs are ubiquitous environmental contaminants. Their accumulation in soil contributes to the further transport of these pollutants into ground water, plants and food (Patel et al., 2020). PAHs pose a serious threat to human health, as they are known to induce oxidative stress, DNA damage, and inflammation, which can lead to various diseases including cancer (Gualtieri et al., 2010; Kang et al., 2010). As this study focuses on the response of yeast cells to PAHs (toxicodynamic) using oxidative stress as biomarker. Thus, the biotransformation of PAHs in yeast (toxicokinetic) has not been conducted. The lack of metabolic study and redox status are the limitations of this study. The response of yeast cells exposed to PAHs from this study is employed to support the results derived from co-culture model of lung cells and macrophages treated with the same chemical set in order to create reference data for health risk assessment (Takam et al., 2024). Therefore, the use of oxidative stress biomarkers conducted in yeast model in this study was found to be a suitable approach for evaluating the toxic effects of PAHs, because of the high sensitivity, cost effectiveness and reproducibility. Furthermore, the oxidative stress biomarkers employed in this study may also be applicable for assessing the toxicity of other environmental xenobiotics.

## 4 Conclusion

This study provides data related to the toxic effect of PAHs with distinct molecular weights on the eukaryote model organism, *S. cerevisiae,* using oxidative stress response biomarkers. The response of yeast cells to PAHs can be identified by both molecular and cellular alterations. For the former, an expression level of genes related to an oxidative stress response was studied. For the latter, ROS formation, SOD activity, and GSH content were quantified. The overall results showed the ultimate toxicity of HMW-PAH caused by the induction of oxidative stress in yeast. This study provides a useful reference for investigating the effects of PAHs on *S. cerevisiae* using oxidative stress biomarkers.

## Data Availability

The original contributions presented in the study are included in the article/Supplementary material, further inquiries can be directed to the corresponding author.
