# Supplementary material for: Oxidative stress response of Saccharomyces cerevisiae exposed to different molecular weight of polycyclic aromatic hydrocarbons
Source: Front Microbiol. 2026 May 25;17:1832345. doi: 10.3389/fmicb.2026.1832345 (PMC13243391; doi:10.3389/fmicb.2026.1832345)
Supplement: Supplementary file 1 [file Data_Sheet_1.PDF]

Supplementary Material

1 Supplementary Figures and Tables

1.1 Supplementary Figures

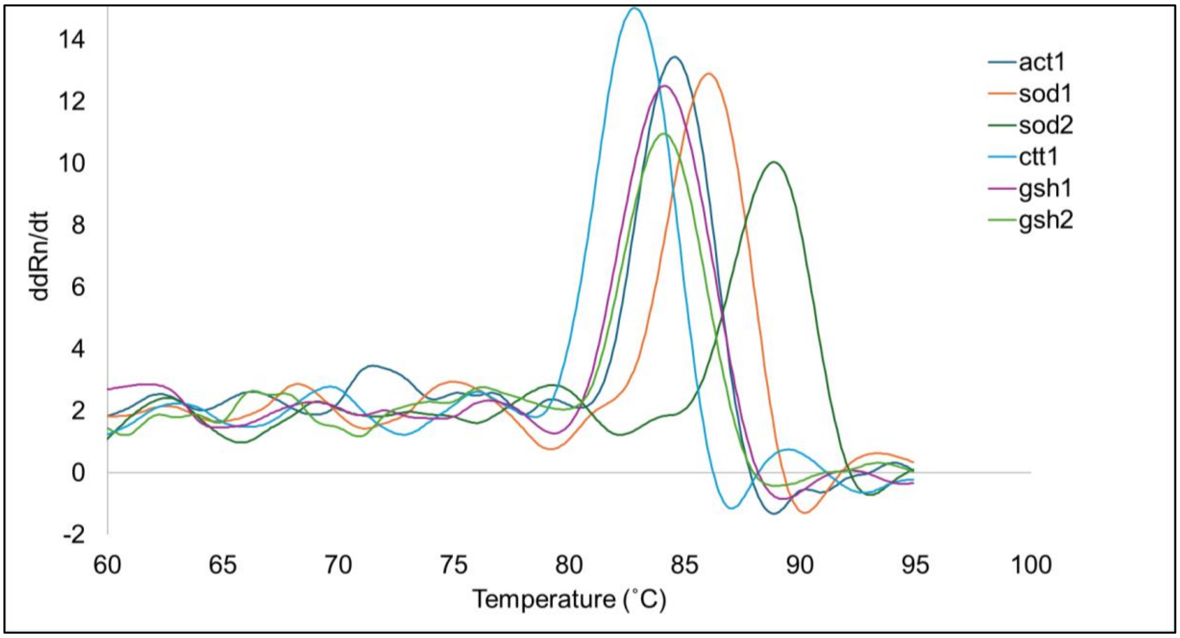

Supplementary Figure 1. Melting curve analysis

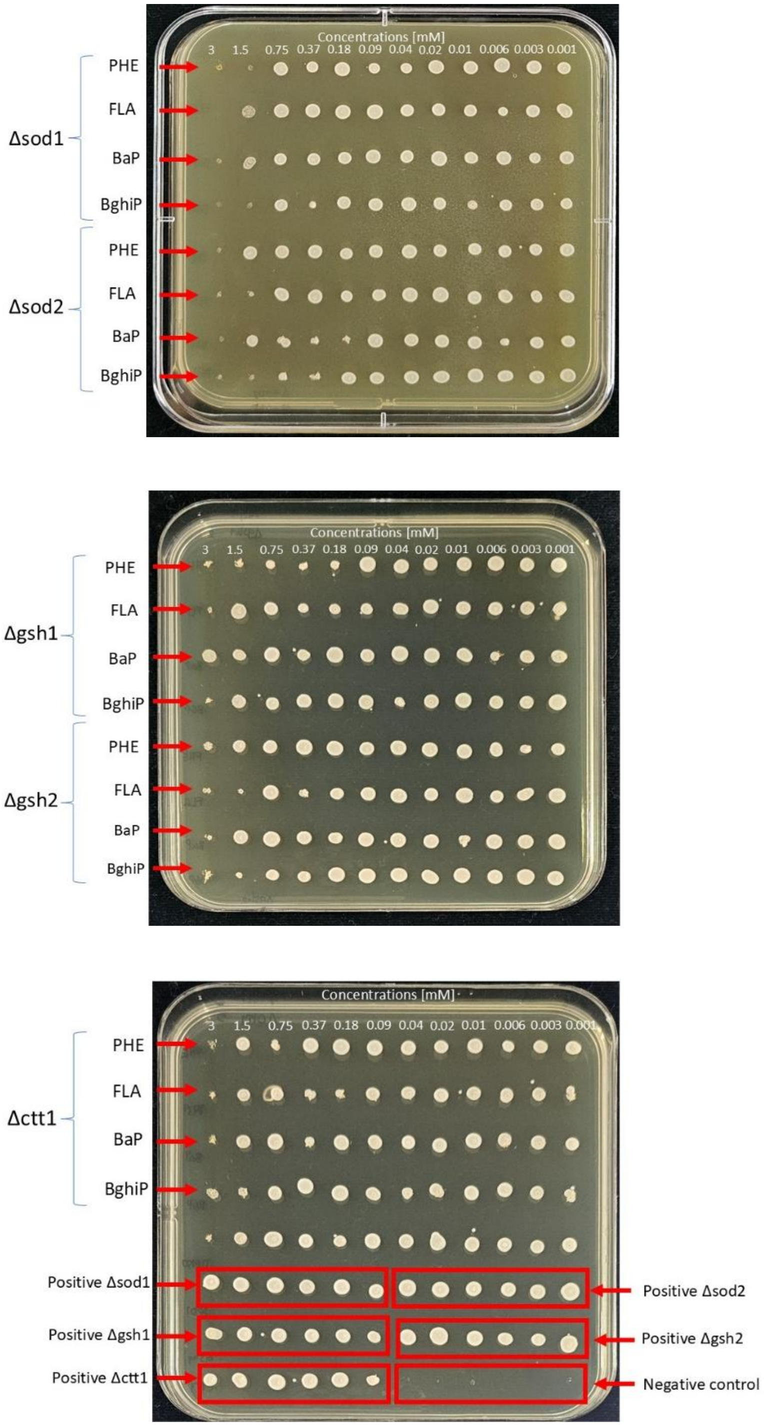

Supplementary Figure 2. Viability of BY4742 and five mutants exposed to different concentrations of PAHs on agar plate.

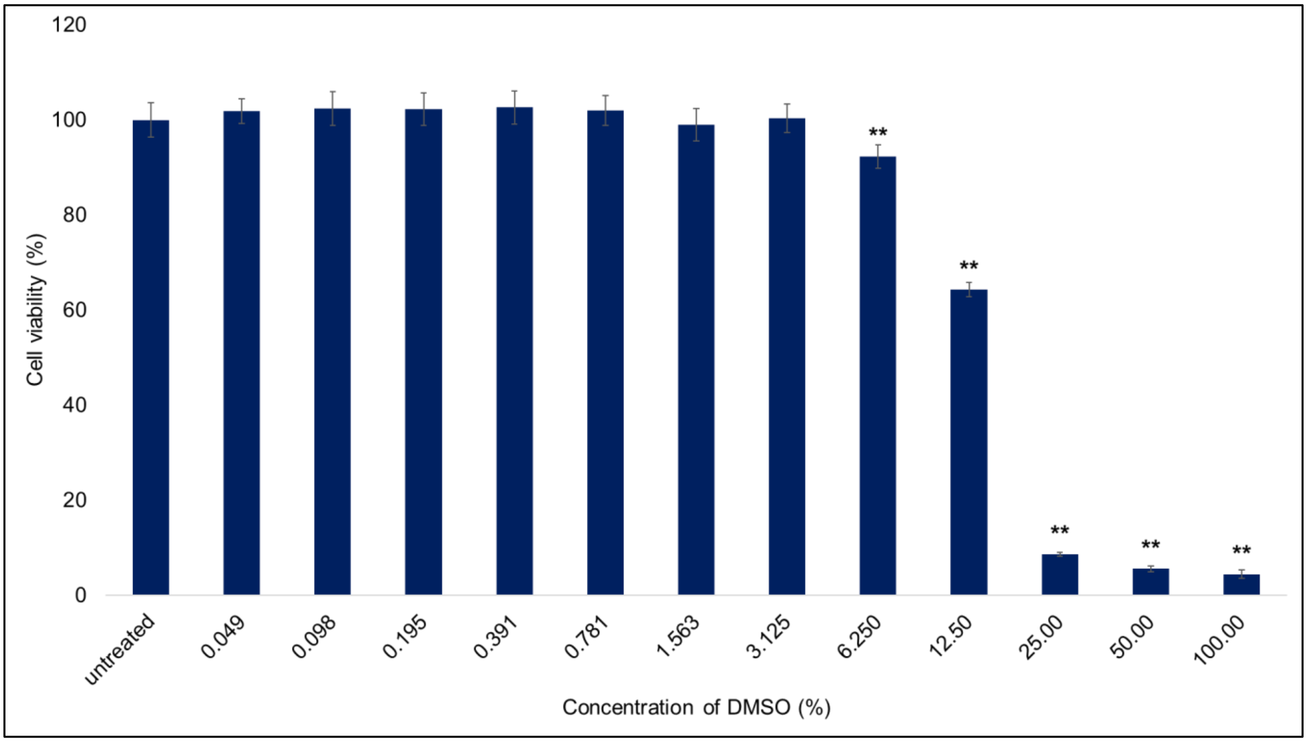

Supplementary Figure 3. The viability of BY4742 exposed to DMSO was assessed. A significant reduction of cell viability was observed at DMSO concentration of 6.25% and above in comparison to the untreated group.

Supplementary Table 1. Oligonucleotide primers and amplicon characteristics were used in RT-qPCR.

primer name	5'-3'	gene targets	product size (bp)	Tm	ref	Amplicon Tm (°C)	Amplification efficiency (%) ±S.D.	R^2
ACT1_F	CCTTCTGTTTTGGGTTTGGA	*Actin*	166	61.5	Babele (2019)	84.35	91.60	0.995
ACT1_R	CGGTGATTTCCTTTTGCATT			60.4				
*SOD1*_F	TTGTAGGCAGAAGCGTCGTT	*Cytosolic superoxide dismutase*	113	59.97	Sillapawattana et al. (2024)	85.85	96.86	0.967
SOD1_R	AATGACACCACAGGCTGGTC			60.25				
SOD2_F	TTCTGGGAAAACCTGGCTCC	*Mitochondrial superoxide dismutase*	105	59.89		89.05	97.24	0.988
SOD2_R	AATCAGCTCGTCCAGACTGC			60.11				
CTT1_F	GATCAATCAGCTCAGCTTCACA	*Catalase*	135	58.73		82.90	97.47	0.892
CTT1_R	GGCCGTCTGGTCTTGAGTAT			59.18				
GSH1_F	GTCTGTCCCTTTGACGCTGA	*γ-glutamylcysteine synthetase*	100	59.97		83.95	101.20	0.970
GSH1_R	AGACCTGGAAGCGGCATTTT			60.25				
GSH2_F	AGAGCAGGAAATAGCGGTGG	*Glutathione synthetase*	120	59.82		84.05	97.96	0.929
GSH2_R	TCTGGGGCCTTTATTGCGAA			59.67				
